# Public Health Consequences on Vulnerable Populations from Acute Chemical Releases

**DOI:** 10.4137/EHI.S828

**Published:** 2008-07-09

**Authors:** Perri Zeitz Ruckart, Maureen F. Orr

**Affiliations:** 1 Agency for Toxic Substances and Disease Registry, Division of Health Studies

**Keywords:** vulnerable populations, chemical release, emergency preparedness

## Abstract

Data from a large, multi-state surveillance system on acute chemical releases were analyzed to describe the type of events that are potentially affecting vulnerable populations (children, elderly and hospitalized patients) in order to better prevent and plan for these types of incidents in the future. During 2003–2005, there were 231 events where vulnerable populations were within ¼ mile of the event and the area of impact was greater than 200 feet from the facility/point of release. Most events occurred on a weekday during times when day care centers or schools were likely to be in session. Equipment failure and human error caused a majority of the releases. Agencies involved in preparing for and responding to chemical emergencies should work with hospitals, nursing homes, day care centers, and schools to develop policies and procedures for initiating appropriate protective measures and managing the medical needs of patients. Chemical emergency response drills should involve the entire community to protect those that may be more susceptible to harm.

## Introduction

Children, elderly, and hospitalized patients may be considered vulnerable populations because they could be more at risk for illness from hazardous substances than other population groups because of their unique characteristics.^1^–^3^ Additionally, if these individuals need assistance in evacuating or implementing other protective measures, then they may be less able to protect themselves from hazards. The Hazardous Substances Emergency Events Surveillance (HSEES) system, initiated by the Agency for Toxic Substances and Disease Registry (ATSDR) in 1990, collects data on acute releases of hazardous substances and associated public health impacts in 14 currently funded states. These data are used in planning and prevention efforts to minimize or prevent releases and their associated adverse public health consequences. HSEES began collecting data on vulnerable populations in 2003 when a geographic information system (GIS) component was added to the data entry system. This analysis describes HSEES events with potential to adversely affect vulnerable populations and suggests measures to prevent and plan for these types of incidents in the future.

## Methods

HSEES is an active, state-based surveillance system that describes the public health impact of the acute releases of hazardous substances and promotes activities to lessen the impact. A HSEES event is defined as an uncontrolled and/or illegal acute release of any hazardous substance meeting specific pre-established criteria. Threatened releases of qualifying amounts of a hazardous substance are included if the threat led to an evacuation or other action to protect the public health. The Petroleum Exclusion clause of the Comprehensive Environmental Response, Compensation, and Liability Act prohibits ATSDR from becoming involved with incidents where any form of petroleum is released that has not been refined to the point of becoming specific chemical products such as pure xylene.^4^ However, HSEES does record information about petroleum if it is released with another qualifying substance.

State health department personnel collect information about the acute hazardous chemical events from a variety of sources (e.g. records and oral reports of state environmental agencies, police and fire departments, and hospitals). Data are entered into a secure Web-based application that enables ATSDR to instantly access data except for identifiable information. Information collected for each event include the location, industry involved, area impacted, proximity to vulnerable populations, chemicals released, number of victims, evacuations, and contributing factors for the event. The HSEES system collects data on the primary (root) contributing factor and the secondary (immediate) contributing factor related to an event. Information on contributing factors is either reported by the notification source or determined by the state HSEES coordinator using various reports.

The 2002 North American Industry Classification System (NAICS) was used to categorize the industries.^5^ Area impacted by the events was defined as where the substance dispersed. GIS or health department records were used to determine possible locations of vulnerable populations (i.e. child day care centers, hospitals, nursing homes, and schools) within close proximity of the event, defined as within a ¼ mile radius. A victim was defined as a person experiencing at least one documented adverse health effect, such as respiratory irritation or chemical burns, which occurred within 24 hours after the release.

For the analyses, the chemicals released were grouped into 13 categories: acids, ammonia, bases, chlorine, hydrocarbons, mixture across categories, oxygenated organics, paints and dyes, pesticides, polymers, volatile organic compounds (VOCs), other inorganic substances, and other substances. Mixture across categories consisted of single chemicals with components from more than one of the other 12 chemical categories The category “other inorganic substances” comprised all inorganic substances—except acids, bases, ammonia, and chlorine—and included chemicals such as nitrogen oxide and hydrogen sulfide. The “other” category consisted of chemicals, such as asbestos, that could not be classified into any of the other 12 chemical categories.

The analysis included events captured by HSEES for 2003–2005. Thirteen states participated in HSEES during the entire period: Colorado, Iowa, Louisiana, Minnesota, Missouri, New Jersey, New York, North Carolina, Oregon, Texas, Utah, Washington, and Wisconsin. An additional four states participated during portions of the period: Alabama (2003); Florida (2005); Michigan (2005); and Mississippi (2003). Analyses were restricted to events where substances were actually released into the environment and the area of impact was >200 feet from the facility/point of release to ensure that there was the potential for an off-site consequence. Events where day care centers, hospitals, nursing homes or schools were recorded within ¼ mile of the event were examined because it would be expected that vulnerable populations were more likely to be found at these locations than elsewhere in the community. Descriptive statistics are presented including time and day of occurrence, duration of the event, contributing factors, chemicals and industries involved in the releases, release type, categories of victims, types of adverse health effects, severity and disposition of the victims, evacuations, in-place sheltering, and personnel who responded to the events.

## Results

During 2003–2005, there were 24,686 HSEES events where at least one substance was actually released. In 231 (0.9%) events there was the potential to affect vulnerable populations because day care centers, hospitals, nursing homes or schools were located within ¼ mile of the event and the area of impact was >200 feet from the facility/point of release. Within ¼ mile of the events were hospitals (10, 4.3%), nursing homes (42, 18.2%), schools (140, 60.6%) and licensed day care centers (146, 63.2%). In most (61.0%) events where the area of impact was >200 feet from the facility/point of release, the area did not extend beyond ¼ mile.

Of the 231 events, 165 (71.4%) occurred in a fixed facility and 66 (28.6%) were transportation-related. When facilities serving vulnerable populations were found within ¼ mile of the event, the most frequent industries involved in the release were manufacturing (59, 25.5%), transportation/warehousing (35, 15.2%), and utilities (34, 14.7%) (Table 1).

### Time and day of occurrence and duration of event

Most (194, 84.0%) of the events occurred on weekdays. For these events, 62.8% occurred during 6:00 am–5:59 pm, 20.1% occurred during 6:00 pm–11:59 Pm, and the remainder occurred during the early morning hours. Time of occurrence was unknown for 2 weekday events. For events occurring on weekends (37, 16.0%), 72.9% occurred during 6:00 am–5:59 pm, 13.5% occurred during 6:00 pm–11:59 Pm, and the remainder occurred during the early morning hours.

Duration of the event was available for 182 (78.8%) events. The duration of the emergency response to the release was from 1–2 hours for 26.4% of the events, less than 1 hour for 15.9% of the events, 2–3 hours for 9.3% of the events, 3–4 hours for 10.4% of the events, and 4 hours or longer for 37.9% of the events. The duration was more than 24 hours for 8.8% of the events.

### Contributing factors

The most frequent primary contributing factors were equipment failure (112, 48.5%) and human error (85, 36.8%). The remaining primary factors were intentional or illegal act (24, 10.4%), bad weather (7, 3.0%), or were missing (3, 1.3%). The most frequent secondary contributing factors were fire (34, 22.4%), unauthorized or improper dumping (25, 16.4%), and improper filling, loading, or packing (21, 13.8%). There was no secondary factor in 79 (34.2%) events.

### Chemicals

A total of 349 chemicals were involved in the 231 events. In five events, one chemical was actually released and one chemical was threatened to be released. The number of chemicals released per event ranged from 1 to 58; however, in most (219, 94.8%) events, only one or two chemicals were released. One or two release types could be reported per substance. Most releases were spills (135, 38.7%), followed by air releases (85, 24.4%), fires (79, 22.6%), spills/fires (22, 6.3%), spills/air releases (14, 4.0%), explosions (3, 0.9%), or other (11, 3.2%).

The top five individual substances released were ammonia (25), carbon monoxide (CO) (23), paint or coating not otherwise specified (NOS) (13), hydrochloric acid (10), and sulfur dioxide (7). The broader categories of substances most frequently released were other inorganic substances (54, 15.5%), oxygenated organics (48, 13.8%), and other (43, 12.3%) (Table 2). Although pesticides, ammonia, and chlorine were not among the most frequently released chemical categories, events involving these chemical categories were the most likely to result in victims (43.8%, 38.5% and 25.0% of all releases in that category, respectively).

### Victims

Adverse health effects were reported by 205 persons in 31 (13.4%) events. Additionally, in 6 (2.6%) events 33 people without symptoms were observed at a medical facility but did not receive treatment. The general public (127, 62.0%) was the population group most frequently injured. Employees (46, 22.4%) and responders (32, 15.6%) were also injured in these events. Respiratory irritation (91, 24.7%) was the most common injury (Table 3). Most (80, 43.2%) victims were 20–44 years of age followed by 5–14 years of age (62, 33.5%) and 45–64 years of age (31, 16.8%); 6 (3.2%) each were 15–19 years of age or 65 or more years of age. The age category was unknown for 20 (9.8%) victims. No victims were reported to be less than 5 years of age. The majority (59.5%) of the victims was male; sex was not identified for 15 (7.3%) victims.

Most victims were treated at a hospital and released (83, 40.5%) or had their injuries reported by an official within 24 hours of the event (66, 32.2%). The other victims were admitted to a hospital (21, 10.2%), were treated on the scene (18, 8.9%), were seen by private physician within 24 hours of the event (9, 4.4%), died as a result of the event (3, 1.4%), were observed at a hospital but did not receive treatment (1, 0.5%), or their disposition was unknown (4, 2.0%). Thirty five of the victims (17%) received decontamination at the scene, a medical facility, or at both locations.

### Evacuations, in-place sheltering, and response

Evacuations were ordered during 64 (27.7%) events. Approximately 47% of the evacuations were of the building where the release occurred, 26.6% were of a circular area surrounding the release, 12.5% were downwind from the release, 10.9% were of both a circular area and downwind of the release, and the remainder used no criteria or were unknown. Durations of the evacuations ranged from 12 minutes to 13 days (median = 2 hours). More than 7,013 people were evacuated during the 64 events (median = 40 people; maximum = 2,000 people per event). Duration of evacuation was unknown for three events, and number of people evacuated was unknown for eight events. Orders to shelter-in-place were issued during 18 (7.8%) events. Information on in-place sheltering was missing for four events.

Fire departments responded to 121 (52.4%) events. Other personnel who frequently responded to the events included the response team of the company where the release occurred (120, 51.9%), law enforcement agency (78, 33.8%), environmental agency/US Environmental Protection Agency (EPA) response team (74, 32.0%), certified HazMat team (60, 26.0%), and emergency medical technicians (43, 18.6%). There was no formal response in 14 (6.1%) events.

### Case vignettes

One employee died from trauma injuries and two members of the general public died from respiratory injuries following a railroad collision caused by human error. The collision caused multiple cars to derail, a puncture in a tank car loaded with chlorine, and a fire in some of the rail cars. This event released 120,000 pounds of chlorine, 78,000 pounds of urea, 7,000 pounds of diesel fuel, and 60,000 pounds of a reaction by-product of chlorine, sodium hydroxide, and sodium hypochlorite. The release impacted an area more than 1 mile from the collision site, and a school was within ¼ mi of the event. There were 41 additional victims in this event: 13 employees, 22 members of the general public, 4 responders (unknown type), and 2 career firefighters. Orders to shelter-in-place were given initially because evacuation was not feasible due to flooding and bridge damage in the immediate area. Subsequently, 45 people were evacuated for 13 days when the company prepared to unload the chlorine car. Age was available for five victims who were members of the general public; but it was known that four of them were 65 or more years of age.

Another event was triggered when a farm worker misapplied a mixture of pesticides causing 67 people at a nearby elementary school to experience adverse health effects including gastrointestinal problems, headaches, dizziness/central nervous system effects, and respiratory and skin irritation. The farm worker used the wrong applicator, and although he allowed a 50 foot buffer zone between the farm and the school, 8–10 mph winds blew the mixture into the school’s air handling system. The school was immediately evacuated and remained closed for one week until clean-up was completed. Fifty-seven (85.1%) victims were students and 8 (11.9%) were staff members; it was unknown if the remaining 2 (3.0%) victims were students or staff members. Most (89.6%) victims had their injuries reported by an official within 24 hours of the event, 6 (9.0%) were seen by a private physician within 24 hours of the event, and 1 (1.5%) was treated at a hospital but not admitted.

In 22 events, CO was released from underground utility cable fires and explosions or was emitted from utility portals (i.e. “manholes”) and drawn into nearby buildings through ventilation systems. The rubber coating of utility cables can crack and split, and subsequent contact with water and road salt from de-icing can cause electrical shorts and underground fires and explosions.^6^ This results in noxious emissions containing CO which travel along conduits under streets and ultimately migrate into indoor environments. Four (18.2%) of the 22 CO events resulted in 5 victims who experienced gastrointestinal problems, headaches, and/or dizziness/central nervous system effects. Evacuations were required in 19 (86.3%) events, and day care centers, nursing homes, and schools were within ¼ mile of 16, 8, and 3 events, respectively.

## Discussion

During 2003–2005, there were 231 events where vulnerable populations were potentially at risk of hazardous substance exposure in HSEES states alone. Hospitals, nursing homes, schools, and day care centers were frequently within ¼ mile of an event when the area of impact was >200 feet (4.3%, 18.2%, 60.6%, and 63.2%, respectively). This does not include many incidents where hospitals, nursing homes, schools and day care centers were the initial location of an event, since many were excluded from this analysis because the substance dispersion did not extend >200 feet from the facility/point of release. Most events occurred on a weekday during times when day care centers or schools were likely to be in session. Releases involving aerosolization, fires, and explosions, which have a greater potential of affecting off-site areas than spills, accounted for approximately 60% of the releases.

Events from 2003–2005 where vulnerable populations were within ¼ mile of the event and the area impacted was >200 feet from the facility/point of release were more likely than all events during this period to result in victims (13.4% vs. 8.3%). Additionally, they were more likely to injure members of the general public (62.0% vs. 36.7%) and more likely to injure persons who were 5–14 years of age (33.5% vs 7.0%). The lack of victims in the younger age groups may imply that the community had appropriate protective measure in place.

There were more orders to shelter-in-place (7.8% vs 1.0%) and evacuate (27.7% vs 5.9%) in these events as well. These differences emphasize that there is more of an impact to the general public in events that are in close proximity to vulnerable populations. However, using the data that is currently collected, we are unable to determine which specific segments of the general public (i.e. nursing home residents, hospital patients) were affected.

Equipment failure and human error caused most of the releases. Substances of particular concern in this analysis were CO (with no known warning properties), chlorine and ammonia (toxic inhalation hazards), and pesticides (due to misapplication). Therefore, facilities that manufacture, use, or store chemicals (particularly those identified in this analysis) and that are in close proximity to vulnerable populations can take standard precautions to prevent equipment- and human error-related releases. Facilities should establish a point of contact with hospitals, nursing homes, day care centers, and schools that are within ¼ mile so they can directly notify them when an event occurs. Operators of these facilities with vulnerable populations should work within their community to be aware of and prepare for likely emergencies.^3^

To reduce hazards to vulnerable populations, communities and facilities should work together. Cutting back emissions, minimizing the quantities of chemicals stored, switching to less hazardous chemicals, better employee training and preventative equipment maintenance are examples of ways that companies can reduce risks and improve efficiency.^3^ The EPA RMP database (which covers facilities with threshold amounts of the most hazardous chemicals on-site) includes data accessible to government officials and designated emergency planners for community response planning. Facilities handling chemicals that could pose a risk to the public (particularly the most vulnerable sectors) have the general duty to identify the hazards of their operations, design and operate safe plants, and be prepared to mitigate any releases that occur.^7^

The information available under the Emergency Planning and Community Right-to-Know Act (EPCRA) and the Clean Air Act can help schools, nursing homes, daycare centers, schools and hospitals prepare for emergencies and identify opportunities to better protect those in their care. Some ways that they can participate are to join the local emergency planning committee (LEPC) when possible. If an LEPC has not been formed in their community, they may wish to encourage its formation. Regardless of whether an LEPC is formed or not, facilities serving vulnerable populations should inform community emergency planners of their existence and location within the community so that appropriate response and protective measures can be included in the community emergency response plan.^7^

Facilities serving vulnerable populations should know how they will be notified in the event of an accident and be prepared to protect those under their care. LEPCs and other community planners such as fire departments and emergency management agencies should know what hazardous chemicals are present in the community and this can help hospitals, schools, day care centers, nursing homes, and similar facilities prepare their internal plans.^7^

The information collected under community right-to- know laws and through HSEES provides land use planners, school boards, property developers, and businesses with data they can use to make informed decisions about placement of new industrial facilities and whether to allow development close to existing facilities that handle hazardous chemicals. Land use planning agencies and others involved in planning decisions should work with the LEPC to develop maps that show where facilities with chemical inventories are located and document those facilities with previous releases. The more likely scenarios and previous incidents should be taken into account when planners are deciding where to locate nursing homes, schools, day care centers, hospitals.^7^

Chemical emergency preparedness and response agencies (e.g. fire departments, emergency management organizations, public health agencies, and law enforcement agencies) should document the location of chemical facilities within their jurisdiction and their proximity to facilities likely to serve vulnerable populations so they can coordinate protective measures to reduce harm and manage the medical needs of patients resulting from acute chemical release incidents. These planning and response agencies should also work with hospitals, nursing homes, day care centers, and schools to develop policies and procedures for evacuation or sheltering-in-place.^8^ The policies and procedures should be communicated to family members of patients, residents, and students to lessen concern.^9^ Institutions housing vulnerable populations should also exercise their staff in their internal procedures and participate in community emergency drills whenever possible.

## Conclusion

Data from HSEES suggests that there is the potential for chemical emergencies to impact facilities serving vulnerable populations. There are a number of ways that these facilities, industries and agencies involved in preparing for and responding to chemical emergencies can proactively work together to minimize the hazards.

## Figures and Tables

**Table 1:** Industries involved in events with vulnerable populations within ¼ mile and the area of impact was beyond the facility/point of release, Hazardous Substances Emergency Events Surveillance, 2002–2005.

**Industry category**	**Frequency**	**Percent**
Agriculture, Forestry, Fishing and Hunting	12	5.2
Construction	10	4.3
Educational Services	8	3.5
Health Care and Social Assistance	5	2.2
Manufacturing	59	25.5
Other*	25	10.8
Retail Trade	8	3.5
Transportation/Warehousing	35	15.2
Utilities	34	14.7
Wholesale Trade	10	4.3
Unknown/Not an industry	25	10.8
Total	231	100.0

* Includes Administrative and Support and Waste Management and Remediation Services (3), Arts, Entertainment, and Recreation (1), Finance and Insurance (1), Information (1), Other Services (13), Public Administration (4), and Real Estate and Rental and Leasing (2).

**Table 2:** Substance categories involved in events with vulnerable populations within ¼ mile and the area of impact was beyond the facility/point of release, Hazardous Substances Emergency Events Surveillance, 2002-2005.

	**Total releases**			**Releases with victims**	

**Substance category**	**No.**	**% of total releases**	**No.**	**% of all releases with victims**	**% of releases with victims in chemical category**
Acids	23	6.6	4	8.3	17.4
Ammonia	26	7.4	10	20.8	38.5
Bases	14	4.0	0	0.0	0.0
Chlorine	8	2.3	2	4.2	25.0
Hydrocarbons	16	4.6	0	0.0	0.0
Mixture across categories	27	7.7	3	6.2	11.1
Other inorganic substances	54	15.5	2	4.2	3.7
Oxygenated organics	48	13.8	4	8.3	8.3
Paints and dyes	16	4.6	0	0.0	0.0
Pesticides/Agricultural	32	9.2	14	29.2	43.8
Polymers	19	5.4	0	0.0	0.0
Volatile organic compounds	23	6.6	5	10.4	21.7
Other*	43	12.3	4	8.3	9.3
Total	349	100.0	48	99.9	NA

* Includes formulations (1), hetero-organics (2), polychlorinated biphenyls (2), indeterminate (2), and other (36).

**Table 3:** Type of injury experienced by victims of events with vulnerable populations within ¼ mile and the area of impact was beyond the facility/point of release, Hazardous Substances Emergency Events Surveillance, 2002–2005.

**Type of injury**	**Frequency**	**Percent**
Burns*	15	4.1
Dizziness/Central nervous system effects	39	10.6
Eye irritation	39	10.6
Gastrointestinal problems	71	19.2
Headache	62	16.8
Heat stress	12	3.3
Other	2	0.5
Respiratory irritation	91	24.7
Shortness of breath	12	3.3
Skin irritation	5	1.4
Trauma^†^	21	5.7
Total	369	100.2

* 14 were thermal burns and 1 was both chemical and thermal burns.

† 12 were chemical-related and 9 were not chemical-related.
